# Editorial: Bone and Cartilage Diseases—The Role and Potential of Natural Products

**DOI:** 10.3389/fphar.2022.938303

**Published:** 2022-05-26

**Authors:** Daohua Xu, Longhuo Wu, Qian Chen

**Affiliations:** ^1^ Guangdong Key Laboratory for Research and Development of Natural Drugs, School of Pharmacy, Guangdong Medical University, Dongguan, China; ^2^ College of Pharmacy, Gannan Medical University, Ganzhou, China; ^3^ Department of Orthopaedics, Warren Alpert Medical School of Brown University/Rhode Island Hospital, Providence, United States

**Keywords:** bone, cartilage, osteoporosis, osteoarthritis, medicinal plants

Traditional medicinal plants and nutraceuticals are often used in the therapeutic management of bone and cartilage diseases, and the secondary metabolites are the main agents responsible for their pharmacological activities. The metabolites provide a great resource for developing new potential drugs.

The pathological mechanisms of bone and cartilage diseases, such as osteoporosis and osteoarthritis, are orchestrated by dysregulated signaling pathways, which disrupt homeostasis by induction of catabolic factors as well as down-regulation of anabolic factors. A large number of studies reveal that many signaling pathways, such as NF-κB pathway, MAPK pathway, TLR pathway, Wnt/β-catenin, TGF-β, and Notch pathways, have been involved in regulating the development of bone and cartilage diseases, and the key factors in these pathways might be potential targets for new drugs development. Thus, understanding the underlying molecular mechanisms offers an avenue for researchers to explore the potential interaction between secondary metabolites and bone and cartilage diseases. The research topic entitled “Bone and Cartilage Diseases—The Role and Potential of Natural Products” included 10 original research, 5 review and 1 clinical trial.

## Osteoporosis

Osteoporosis is a common metabolic bone disease, including bone loss due to postmenopausal, senile, and secondary osteoporosis. Although there are many therapeutic drugs available for osteoporosis treatment including bisphosphonates, calcitonin, and parathyroid hormone, the long-term use of these drugs may produce inherent side effects. It is imperative to search for alternative natural compounds (Wang et al., 2017).

Han et al. showed that E.C.D. extracts, consisting of *Eucommia ulmoides* Oliv., *Cuscuta chinensis* Lam, *Davallia trichomanoides* Blume, decreased the loss of urinal calcium and phosphorus and the expression of RANKL and C-terminal telopeptides of type l collagen (CTX) in rat serum. In addition, E.C.D. extracts increased the levels of serum calcium, phosphorus, and osteoprotegerin in experimental glucocorticoid-induced osteoporosis (GIO) rats. E.C.D. extracts also improved bone density, structural integrity, and biomechanical function in experimental GIO rats. The mechanisms of E.C.D. extracts against osteoporosis may be associated with the inhibition of osteoclast differentiation through PI3K/Akt pathway.

The study by Lee et al. showed that *Sparganii Rhizoma* protected against bone loss in ovariectomy rats. *Sparganii Rhizoma* inhibited osteoclast differentiation and bone resorption by down-regulating the activity of NF-κB and MAPK pathways. *Sparganii Rhizoma* also stimulated osteoblast differentiation by increasing the expression of bone morphogenetic protein 2 (BMP-2)/SMAD pathway. Mogrol treatment reduced the bone loss in ovariectomy mice and inhibited the differentiation of osteoclasts by mediating NF-κB and MAPK signaling pathways. It suggested that Mogrol might be a promising compound for preventing or treating osteoporosis (Chen et al.).

The traditional Chinese medicine herbal extracts (Jing extracts) includes *Astragalus mongholicus* Bunge, *Cistanche deserticola* Y.C.Ma, *Dioscorea polystachya* Turcz., *Lycium barbarum* L., *Epimedium brevicornu* Maxim, *Cinnamomum cassia* (L.) J. Presl, *Syzygium aromaticum* Merr. and L.M.Perry, *Angelica sinensis* (Oliv.) Diels, and *Curculigo orchioides* Gaertn. The original research by Qian et al. showed that the Jing extracts alleviated alcohol-induced bone loss by micro-CT analysis of the proximal tibia of male mice. The Jing extracts prevented chronic excessive alcohol consumption-induced osteopenia in male mice.

Diabetic osteoporosis is a disorder of bone metabolism, which is caused by long-term exposure to high glucose and impairment of osteoblast function. Xu et al. showed DAI-1 was a polysaccharide extracted from *Dipsacus asperoides*, which could reverse the inhibition of MC3T3-E1 cell proliferation and differentiation by high levels of glucose. The mechanism is through mediating the activity of BMP-2/Smad/Runx2 signaling pathway.

Anthocyanins are a class of naturally water-soluble flavonoid compounds obtained from colored plants. Mao et al. reviewed the effects of anthocyanins on bone regeneration and their molecular mechanisms. This review showed that anthocyanins promoted osteogenesis by the BMP2 pathway, Wnt/β-catenin pathway, and FGF pathway. In addition, anthocyanins inhibited osteoclastogenesis by the MAPK pathway, NF-κB pathway, and Ca^2+^ pathway.

Bone marrow mesenchymal stem cells (MSCs) are multipotent, which may have the rapeutic potential for bone related diseases. Zhang et al. reviewed the roles of flavonoids in the osteogenic differentiation of MSCs. Flavonoids promoted proliferation and osteogenic differentiation of MSCs and regulated the microenvironment in damaged bone. Combination of MSCs with flavonoids may be a promising alternative to stem cell therapy alone.

## Fracture

Bone is one of the organs with regenerative ability. Fracture repair usually restores a damaged bone to its pre-injury state. However, approximately 10% of fractures do not heal properly (Einhorn and Gerstenfeld, 2015). It is important to develop drugs for fracture healing. Ginsenoside compound K improved fracture repair in rat open femoral fracture model (Ding et al.). Mechanically, Ginsenoside compound K promoted osteogenic differentiation of bone marrow mesenchymal stem cells through activating Wnt/β-catenin signaling pathway. In addition, Ginsenoside compound K increased the tube formation capacity of human umbilical vein endothelial cells (HUVECs) and angiogenesis during fracture healing.

## Osteonecrosis of Femoral Head

Osteonecrosis of femoral head (ONFH), a progressive hip joint disorder, is associated with impaired blood supply to the femoral head. The pathogenesis mechanism of ONFH is not fully elucidated (Rezus et al., 2021). Peng et al. found that serum amyloid A (SAA), an acute phase lipophilic protein, increased significantly in the serum of ONFH patients. SAA inhibited osteogenic differentiation and promoted the adipogenic differentiation of MSCs. *In vivo* results showed that SAA reduced new bone formation. Thus, SAA is a vital protein in the physiological process of ONFH and may be a potential therapeutic target. Huo Xue Tong Luo (HXTL) capsule is composed of seven herbs, including Cajan leaf, *Angelica sinensis* (Oliv.) Diels, *Paeonia lactiflora* Pall, *Ligusticum striatum* DC, *Prunus persica* (L.) Batsch, *Carthamus tinctorius* L, and *Rehmannia glutinosa* (Gaertn.) DC. A clinical study by Peng et al. showed that HXTL capsules improved hip function and delayed progression to femoral head collapse in ONFH of Association Research Circulation Osseous (ARCO) stage II. HXTL capsule was effective and ideally used when the anterior and lateral portions of the femoral head were not affected.

## Osteoarthritis

Osteoarthritis (OA) is a progressive arthrosis disease characterized by subchondral bone damage, synovitis, and articular cartilage degradation. More than 10% of people under the age of 60 worldwide suffers from OA (Runhaar et al., 2015). In this research topic, three reviews and two original studies focused on OA. Phytochemicals for OA treatment were reviewed by Tian et al., who demonstrated that many phytochemicals exhibited therapeutic effects against OA by mediating relevant autophagy-related pathways. Another review by Mu et al. summarized the roles of botanical drug extracts (BDEs) combining with biomaterial carriers to treat OA in recent 10 years. BDEs, containing flavonoids, polyphenols, alkaloids, and saponins, improved joint functions, promoted repair of damaged cartilage, and delayed progression of OA. The combination of BDEs with biomaterial carriers can relieve OA symptoms and delay the progression of OA. Vafaei et al. reviewed the effects of crocin on bone and cartilage diseases. Specifically, crocin has therapeutic potentials on OA, rheumatoid arthritis, and osteoporosis. Crocin reduced oxidative stress and inflammatory responses, promoted the osteogenic differentiation of MSCs, and suppressed osteoclast functions.

Zhuang et al. demonstrated that Jintiange capsule, a traditional medicine composed of various animal bones, organic compounds, and polysaccharides, reduced the subchondral bone remodeling and cartilage degeneration in OA mouse models. The mechanism of Jintiange capsule in the treatment of OA is related to its inhibition of osteoclast activation and chondrocyte apoptosis. The extracellular matrix (ECM) is an important constituent of cartilage (Rahmati et al., 2017). Zheng et al. showed that xanthohumol is a naturally occurring prenylflavonoid derived from hops and beer and inhibited the mechanical stimulation-induced ECM degradation by mediating the GAS5/miR-27a signaling pathway in OA chondrocytes.

## Rheumatoid Arthritis

Rheumatoid arthritis (RA) is an autoimmune disease characterized by joint inflammation, followed by cartilage destruction and bone erosion (Jang et al., 2022). The study by Chen et al. demonstrated that kefir peptides alleviated collagen-induced arthritis in the mouse models by inhibiting dendritic cells maturation and inflammatory cytokine release. The kefir peptides may be potential for the clinical use in RA patients.

## Conclusion

In conclusion, the research topic comprising a collection of 16 articles presents the roles of many natural products for treatment of bone and cartilage diseases. The natural products have shown great prospects for the development of new drugs, which may become new and/or alternative treatment for bone and cartilage diseases. However, randomized, controlled, and double-blind clinical trials of natural products for the treatment of bone and cartilage diseases are needed. Since the roles of natural products could be multi-component and multi-targeted, modern analytical methods in genomics, proteomics, and metabolomics should be applied to unravel their mechanisms of action in treatment of bone and cartilage diseases in the future.
